# Supplementary material for: Implicit sensorimotor adaptation comprises distinct mechanisms for action selection and execution
Source: PLoS Biol. 2026 Oct 9;24(10):e3004057. doi: 10.1371/journal.pbio.3004057 (PMC13667926; doi:10.1371/journal.pbio.3004057)
Supplement: Supplementary file 2 [file pbio.3004057.s001.docx]

**S1 Text. Supplementary results**

Supplementary Result 1: 1.5s delayed feedback sufficiently suppressed implicit recalibration.

We conducted a control experiment (Exp S1) to confirm that the aftereffect observed in Exp 1 was not due to implicit recalibration. For this experiment, we used the same perturbation schedule but replaced the hand-contingent feedback with task-irrelevant feedback (“error clamp”) and instructed participants to always reach directly to the target. If the cursor feedback is not delayed, participants gradually change their reaching direction without awareness in response to clamped feedback [1–3]. However, we delayed the clamped feedback by 1.5 s in this control condition, a manipulation that effectively nullifies implicit recalibration [4,5]. Consistent with this assumption, we did not observe any change in movement direction during the perturbation, nor was there a measurable aftereffect in the no-feedback blocks (Fig 2A). By inference, the absence of an aftereffect in this control experiment indicates that the aftereffects observed in the main experiment reflect the operation of a distinct implicit process. Indeed, a direct comparison of the aftereffect data showed that the group receiving contingent (task-relevant) feedback exhibited larger aftereffects compared to the control group (t(36)=3.7, p<0.001). Interestingly, the absence of an aftereffect in the delayed clamp condition suggests that implicit aiming does not operate when participants have no intention to learn.

# Supplementary Result 2: Implicit aiming is not caused by use-dependent learning

We have hypothesized that the aftereffect observed following the gradual perturbations in Exp 1 reflects the operation of an implicit process contributing to action selection. However, it is possible that the effect is a manifestation of use-dependent learning, a process whereby a movement is biased in the direction of recently repeated movements [6,7]. The magnitude of use-dependent learning in visuomotor adaptation studies is around 2-5° [8,9], similar to the aftereffect values observed in the two probe blocks following the abrupt, 70° rotation. However, the aftereffect values in three probe blocks during the gradual rotation phase were much larger (>10°), suggesting they are unlikely to be caused by use-dependent learning.

Nevertheless, we conducted a control experiment (Exp S2) to ask if these aftereffects were due to use-dependent learning. For this experiment, we ramped up the perturbation to 20° and then ramped it back down to 15° before a no-feedback block to assess the aftereffect. During this no-feedback block, the first five trials participants went to the training location. In the next 40 trials, the target randomly appeared at two alternative positions that flanked the target position by 20° to assess generalization (S1B Fig). If the aftereffect is due to use-dependent learning, we should observe a bias towards the training target; as such, the aftereffect will be in opposite directions for the two displaced targets. In contrast, if the aftereffect is due to implicit aiming, we would expect the bias will be in the same direction for all three targets.

Participants tracked the gradual rotation during the perturbation phase and, consistent with Exp 1, there was a significant aftereffect during the no-feedback block. Importantly, the aftereffect was in the same direction for all three targets, with the magnitude reduced for the two generalization targets. These data are consistent with the hypothesis that the aftereffect is due to an implicit change in aiming rather than a manifestation of use-dependent learning.

Supplementary Result 3: The CPC model failed to predict the learning dynamics with delayed feedback

We simulated the CPC model for the delayed feedback conditions in Exp 3 and 4. For Exp 3, the CPC prediction was nearly identical to that of the clamp condition: The model again predicted an anti-savings effect rather than a savings effect (S4A Fig), at odds with what was observed in the implicit aiming condition of Exp 3. For Exp 4, the CPC model also predicted that the magnitude of the decrease would be reduced across the three breaks, similar to what was observed in the implicit aiming condition. This occurs because the contribution of the volatile process diminishes when the remaining error becomes smaller (S4B Fig). However, the magnitude of the predicted drop was significantly smaller than what was observed in the experiment.

To further compare the CPC and COIN models in predicting the temporal stability of implicit aiming, we conducted an additional experiment (Exp S3). This experiment replicated the design of Exp 4 but included a long baseline block with feedback before the perturbation training (S5A Fig). According to the COIN model, this extended block will strengthen the prior for the baseline context. As such, after each probe, participants will be more likely to infer that they are more likely to be in the baseline context and the drop in hand angle should be larger after the 1-min breaks compared to when there was no baseline as in the main experiment (S5B Fig). In contrast, the CPC model lacks contextual inference and therefore predicts an identical drop in both the long and no-baseline conditions.

Consistent with the COIN prediction, we found that including a baseline block increased the magnitude of the drop compared to the no-baseline condition of Exp 4 (S5B Fig, linear mixed model, t(161)=3.5, p_FDR_=0.001). This effect cannot be captured by the CPC model. These findings provide additional evidence that the drop after the break in the delayed feedback condition reflects contextual uncertainty, consistent with the COIN model, rather than forgetting of a volatile process as posited by the CPC model. In contrast, for the clamped feedback condition, when 0° clamped feedback (i.e., cursor moves directly to target) was used in an extended baseline block (S5C and S5D Fig), the size of the drop remained the same as in the non-baseline condition (t(269)=-0.56, p_FDR_=0.58), consistent with the prediction of the CPC model.

**References**

1.	Morehead, J. R., Taylor, J. A., Parvin, D. E. & Ivry, R. B. Characteristics of Implicit Sensorimotor Adaptation Revealed by Task-irrelevant Clamped Feedback. *J. Cogn. Neurosci.* **29**, 1061–1074 (2017).

2.	Kim, H. E., Morehead, J. R., Parvin, D. E., Moazzezi, R. & Ivry, R. B. Invariant errors reveal limitations in motor correction rather than constraints on error sensitivity. *Commun Biol* **1**, 19 (2018).

3.	Tsay, J. S., Parvin, D. E. & Ivry, R. B. Continuous reports of sensed hand position during sensorimotor adaptation. *J. Neurophysiol.* **124**, 1122–1130 (2020).

4.	McDougle, S. D. & Taylor, J. A. Dissociable cognitive strategies for sensorimotor learning. *Nat. Commun.* **10**, 40 (2019).

5.	Brudner, S. N., Kethidi, N., Graeupner, D., Ivry, R. B. & Taylor, J. A. Delayed feedback during sensorimotor learning selectively disrupts adaptation but not strategy use. *J. Neurophysiol.* **115**, 1499–1511 (2016).

6.	Diedrichsen, J., White, O., Newman, D. & Lally, N. Use-dependent and error-based learning of motor behaviors. *J. Neurosci.* **30**, 5159–5166 (2010).

7.	Verstynen, T. & Sabes, P. N. How Each Movement Changes the Next: An Experimental and Theoretical Study of Fast Adaptive Priors in Reaching. *Journal of Neuroscience* **31**, 10050–10059 (2011).

8.	Marinovic, W., Poh, E., de Rugy, A. & Carroll, T. J. Action history influences subsequent movement via two distinct processes. *Elife* **6**, (2017).

9.	Tsay, J. S. *et al.* Dissociable use-dependent processes for volitional goal-directed reaching. *Proc. Biol. Sci.* **289**, 20220415 (2022).
