# Supplementary material for: Implicit sensorimotor adaptation comprises distinct mechanisms for action selection and execution
Source: PLoS Biol. 2026 Oct 9;24(10):e3004057. doi: 10.1371/journal.pbio.3004057 (PMC13667926; doi:10.1371/journal.pbio.3004057)
Supplement: Supplementary file 3 [file pbio.3004057.s008.docx]

**S1 Table. Participant demographic information.**

| **Experiment** | **1** | **S1** | **S2** | **2 Implicit** | **2 Explicit** | **3 Delayed** | **3 Clamp** | **4 Delayed** | **4 Clamp** | **S3 Delayed** | **S3 Clamp** |
| --- | --- | --- | --- | --- | --- | --- | --- | --- | --- | --- | --- |
| **Total** | 19 | 19 | 21 | 18 | 20 | 13 | 30 | 30 | 60 | 28 | 34 |
| **Female** | 7 | 11 | 14 | 13 | 14 | 8 | 16 | 16 | 30 | 9 | 15 |
| **Age (SD)** | 22.2(4.5) | 20.0(2.0) | 21.2(2.5) | 22.0(5.1) | 21.7(3.5) | 21.8(2.1) | 28.1(4.7) | 21.4(5.3) | 29.5(3.4) | 21.6(2.9) | 27.6(4.7) |
| **CCW^a^** | 10 | 10 | 11 | 9 | 10 | 7 | 14 | 13 | 27 | 17 | 17 |

^a^: Number of participants who experienced a rotation in the counterclockwise direction during the main perturbation block. The other participants experienced a rotation in the clockwise direction.
