# Supplementary material for: Implicit sensorimotor adaptation comprises distinct mechanisms for action selection and execution
Source: PLoS Biol. 2026 Oct 9;24(10):e3004057. doi: 10.1371/journal.pbio.3004057 (PMC13667926; doi:10.1371/journal.pbio.3004057)
Supplement: Supplementary file 4 [file pbio.3004057.s009.docx]

**S1 Appendix. Instructions for Experiments 1–2**

The point of this experiment is to better understand how the brain controls reaching movements. We are especially interested in how motor control is impacted by various neurological disorders, like Parkinson's disease and cerebellar degeneration. Because you are a healthy student, your data will be used as a normative baseline for future comparisons with these neurological patients. The information we gain from this experiment could help improve rehabilitation for these patients, so please try your best to pay attention and follow all instructions.

You will be holding this 'pen' at the red base and should maintain the same grip throughout the experiment (Demonstrate how to hold the pen). You will be making fast center-out reaching movements towards a blue target. (Physically demonstrate the reaching movements)

Try your best to reach quickly and accurately in a straight line, and try to slice through the blue targets rather than stopping at the target. These are center-out reaches, which means that every trial starts from the exact same home location. You will NOT see your hand during the movement.

Please adjust the seat height and scoot close to the workstation since you will be making many reaches towards the end of the tablet. I want you to be able to do that without moving any parts of your body other than your arm. (Participant gets situated). Try to keep the same posture throughout the experiment. You can see the current number on the upper left. If any of my instructions are unclear, please ask me immediately.

(Start program, move mouse and keypad; turn off lights and close door)

Trial 0 (Baseline): Please slice through the target as precisely as you can.

Trial 20 (Veridical feedback): Good job. From now on, the white dot will inform you of your hand position. Please correct your movement based on the white dot. Please remember to move fast.

Trial 40 (Demonstration block): This time there will be a discrepancy between the white dot and your actual hand movement that will remain the same. Please adjust your actual hand movement to make the white dot appear on the blue target.

Trial 60 (Aftereffect): Now the white dot will disappear. Please resume your reaches directly to the blue target. If you were aiming away from the target to make the white dot hit the target, stop doing that and aim directly at the blue target.

Trial 75 (Veridical feedback): The white dot will appear again. Adjust your movement based on the white dot.

Trial 85 (Demonstration block): This time there will be a fixed discrepancy between the white dot and your actual hand movement that will remain the same; please adjust your actual hand movement to make the white dot appear on the blue target.

Trial 105 (Aftereffect): Now the white dot will disappear. Please resume your reaches directly to the blue target. If you were aiming away from the target to make the white dot hit the target, stop doing that and aim directly at the blue target.

Trial 120 (Gradual rotation):

Exp 1: This time there may or may not be a discrepancy between the white dot and your actual hand movement; please adjust your actual hand movement to make the white dot appear on the blue target.

Prime Implicit: This time there may or may not be a discrepancy between the white dot and your actual hand movement. Importantly, we note that when people reach without seeing their hand, previous studies have shown that their perception of the hand will gradually drift away. You may experience this during the experiment. Please remember we want to adjust your movement to make the white dot appear on the blue target.

Prime Explicit: In the next part of the experiment, you will experience a gradual rotation. At the beginning, the discrepancy between the cursor and your hand will be 0, so you will reach directly towards the blue target, and later the discrepancy will gradually INCREASE to 25 degrees, so please adjust your actual hand movement so the white dot lands on the blue target.

Trial 180 (Aftereffect): Now the white dot will disappear. Please resume your reaches directly to the blue target. If you were aiming away from the target to make the white dot hit the target, stop doing that and aim directly at the blue target.

Trial 195 (Gradual rotation):

Exp 1: The white dot will appear again. Adjust your movement based on the white dot.

Prime Implicit: The white dot will appear again. Adjust your movement based on the white dot.

Prime Explicit: The white dot will appear again. Adjust your movement based on the white dot. The discrepancy between the cursor and your hand starts at 25 degrees, and later the discrepancy will gradually INCREASE to 70 degrees, so please adjust your actual hand movement so the white dot lands on the blue target.

Trial 286 (Aftereffect): Now the white dot will disappear. Please resume your reaches directly to the blue target. If you were aiming away from the target to make the white dot hit the target, stop doing that and aim directly at the blue target.

Trial 301 (Gradual rotation):

Exp 1: The white dot will appear again. Adjust your movement based on the white dot.

Prime Implicit: The white dot will appear again. Adjust your movement based on the white dot.

Prime Explicit: The white dot will appear again. Adjust your movement based on the white dot. At the beginning, the discrepancy will be 70 degrees and later in the next 100 trials, the discrepancy will gradually DECREASE to 25 degrees, so please adjust your actual hand movement so the white dot lands on the blue target.

Trial 392 (Aftereffect): Now the white dot will disappear. Please resume your reaches directly to the blue target. If you were aiming away from the target to make the white dot hit the target, stop doing that and aim directly at the blue target.
