# Supplementary material for: Implicit sensorimotor adaptation comprises distinct mechanisms for action selection and execution
Source: PLoS Biol. 2026 Oct 9;24(10):e3004057. doi: 10.1371/journal.pbio.3004057 (PMC13667926; doi:10.1371/journal.pbio.3004057)
Supplement: Supplementary file 5 [file pbio.3004057.s011.docx]

**Response letter**

Editor remarks:

Thank you for your patience while your manuscript "Distinct Implicit Contributions to Action Selection and Action Execution in Sensorimotor Adaptation" was peer-reviewed at PLOS Biology. Your study has now been assessed and discussed by the PLOS Biology editors, an Academic Editor with relevant expertise and several independent reviewers. As you will see in their reports at the end of this email, despite noting the interest of the study, the reviewers raise significant concerns about the conclusiveness of the work.

Based on these reviews, I regret that we will not be able to invite revision of the study, as successfully addressing the concerns raised would entail a significant amount of work (beyond what could be reasonably expected of a revision) and whether the current conclusions would be supported is unclear. 

You will see that the reviewers think that the study provides potentially important insights. However, R1 and R2 in particular raise concerns about the experiments 1 and 2 being not sufficiently conclusive at the moment, and experiment 3 lacking an important control with a longer washout.  

Because producing the required data would require substantial cost and effort with an uncertain outcome, we cannot in good faith invite revision of your study. However, given our and the reviewers' interest in the topic, if further work were to allow you to comprehensively address the reviewer concerns outlined above, we would be willing to consider an extensively revised manuscript as a re-submission. The article would receive a new number and submission date and we would evaluate it in view of any related work published in the interim (applying our 6-month scooping protection policy, of course), but we would make every attempt to engage the same Academic Editor and reviewers in its assessment, and the process would not start from scratch but continue from here.

We thank the reviewers and the editor for their constructive feedback, and we are pleased to see that all reviewers found the topic interesting and important. Building on their recommendations and your charge letter, we have undertaken a thorough revision of the manuscript featuring the following key changes:

1. In response to Reviewer 3, we ran a new experiment, following the design of Experiment 3 but with an extended washout period to ensure that behavior returned to baseline performance. Critically, this new experiment closely replicated all of the key results of the original experiment. In particular, we observed prominent learning when we limited RT in combination with delayed feedback, as well as a clear savings effect during relearning. While we considered adding the experiment, providing a replication, we decided to just replace the original Experiment 3 with this new study since they are, in effect, the same experiment with the improved methodological addition suggested by Reviewer 3.

2. We have thoroughly addressed the concerns regarding Experiments 1 and 2 raised by Reviewers 1 and 3. We conducted detailed analyses of the alternative hypotheses noted by the reviewers and believe these analyses make a strong case that none of them can comprehensively account for the results. We also added new analyses examining both the switching dynamics and the retention rate observed in the aftereffect, providing additional evidence that implicit aiming exhibits unique properties not observed with other learning processes. Our analyses here also bolster the argument that implicit aiming is NOT an artifact of some participants failing to understand the instructions. These new analyses and clarifications have been incorporated into the manuscript.

3. While Reviewer 1 suggested using error size to manipulate the credit assignment process, we note that this manipulation is part of the design of Experiment 1. As such, we have addressed this issue in our re-analysis of Experiment 1.

In summary, we believe that the new data and analyses incorporated in the revised manuscript substantially strengthen the submission. We provide point-by-point responses to the reviewers’ comments below.

**Reviewer remarks:
Reviewer #1:** The manuscript aims to demonstrate that motor adaptation comprises a previously unidentified implicit learning component—termed implicit aiming—which pertains to action selection rather than action execution. Across four experiments, the authors progressively characterize this component. Experiment 1 combines gradual perturbations with delayed feedback and reports that participants show consistent deviations in no-feedback probe trials, interpreted as an implicit learning process distinct from implicit recalibration. Experiment 2 uses the same paradigm but manipulates task instructions to bias credit assignment toward external or internal sources of error. The authors find that implicit aiming increases after internal priming and decreases after external priming, which they interpret as evidence for its dependence on causal attribution. Experiment 3 employs limited preparation time and delayed feedback and reports savings during relearning, whereas an analogous error-clamp condition shows attenuated relearning. This dissociation is used to argue that implicit aiming is computationally distinct from implicit recalibration. Experiment 4 introduces an abrupt rotation followed by a gradual reduction, with inserted rest breaks, and shows different temporal decay patterns for implicit aiming versus implicit recalibration. Finally, the authors present simulations showing that a contextual-inference (COIN) model captures the properties of implicit aiming in Exp3 and 4, while a population-coding (CPC) model captures the properties of implicit recalibration.

The core empirical observation across these studies is that delayed feedback in a visuomotor rotation task elicits a robust implicit hand deviation when participants are instructed to aim directly at the target. The authors argue that this learning process requires intention, exhibits savings, is modulated by contextual cues, and is captured by a contextual-inference framework. They therefore conceptualize implicit aiming as a covert action-selection adjustment arising from credit assignment of error to internal biases.

I am impressed by the study's richness of new behavioral findings, and the authors also did a great job performing control experiments to clarify some confounds. However, my main concern is that the phenomenon labeled as implicit aiming may reflect residual explicit or cognitive processes that manifest in probe trials or trials with shortened RT, rather than a distinct implicit action-selection mechanism. In line with Occam's razor, I am not convinced that the observed behavioral effect represents a genuinely novel learning component. I listed my reasoning below:

We appreciate the reviewer's summary comments and alternative hypotheses. Before we address your alternative account directly (below), we would like to clarify several general points regarding our conceptualization of implicit aiming:

First, we do not consider implicit aiming to be a fully independent process from explicit aiming. We propose that both processes arise from a common inference process, one in which the action selection system compensates for a consistent failure to reach the target. This requires assigning the error to two potential sources: external perturbations and internal biases. We probe the latter by instructing the participants to aim directly towards the target (when feedback is removed), thus eliminating the aiming strategy adopted to compensate for the external perturbation. The residuals (aftereffect) are what we attribute to implicit aiming.

Second, as we hope we make clear in the paper, recognizing the distinction between explicit aiming and implicit aiming is the novel contribution of our work, one that has been unappreciated and can help account for some puzzles in the literature. While implicit learning was historically treated as a unitary process, various implicit processes are now recognized as distinct; for example, recalibration, some forms of reinforcement learning, and use-dependent learning. But even with this multiplicity of learning processes, there remains seemingly contradictory findings regarding the properties of implicit learning processes. Here, we introduce a new implicit process and, as exemplified in Exps 3 and 4, show how this process is quite different from implicit recalibration, motivating in part our revised taxonomy. We believe this new framework will be very useful for future work designed to understand the properties and origins of different learning processes, moving towards a more comprehensive view of sensorimotor learning.

Third, we propose a mechanistic account of how a single common credit assignment process gives rise to both implicit and explicit aiming. This account provides a coherent interpretation across all experiments which is particularly notable given the different behavioral tasks and probes used in the paper.

To preview, while we can see that some of reviewer's alternative hypotheses are consistent with particular results when considered in isolation, our take is that the implicit aiming hypothesis provides the most parsimonious account of the full set of data.

1. Similarity between implicit aiming and explicit re-aiming
The defining characteristics attributed to implicit aiming—dependence on intention, contextual sensitivity, and coexistence with explicit re-aiming—mirror known properties of explicit learning. Table 1 lists characteristics ostensibly distinguishing implicit aiming, yet all are compatible with explicit re-aiming. The fact that implicit aiming only emerges when explicit aiming is present strongly suggests a shared origin.

As noted above, we agree with the reviewer that implicit and explicit aiming likely share the same origin. This is consistent with the taxonomy we propose where both implicit and explicit aiming are part of the action execution branch. Moreover, as developed in Exp 2, we assume that both processes involve a common inference process with explicit aiming modified based on the component attributed to an inferred external perturbation and implicit aiming modified based on the component attributed to an inferred change in internal state (e.g., motor bias). This highlights one core distinction between explicit and implicit aiming, an idea that has not been discussed in previous work on sensorimotor adaptation.

We have revised the text to emphasize this point (Lines 190-198):

*“Third, the aftereffects in Exp 1 may arise from confusion created in attributing the observed error to a change in the external environment or internal motor biases(Berniker & Kording, 2008). The former would correspond to the participant’s estimate of the experimenter-imposed perturbation; the latter would correspond to the participant’s sense that some of the error is due to idiosyncratic motor biases(Kim et al., 2025a; Wang et al., 2024) and proprioceptive drift that occurs in the absence of veridical feedback(Block & Bastian, 2011; Wann & Ibrahim, 1992). … In other words, explicit and implicit aiming can be viewed as two outputs of a common credit assignment process.”*

2. Interpretation of Experiments 1-2 and alternative explanations
The strong support for implicit aiming comes from Experiments 1 and 2, where exclusion trials following gradual rotation with delayed feedback show systematic deviations, whereas delayed error-clamp exposure produces none. Instructional priming shifts the magnitude of these deviations.
However, these findings can be explained without positing a new implicit action-selection component:
(a) Difficulty fully suppressing explicit re-aiming
In gradual rotation paradigms, participants inevitably start to increasingly engage in explicit re-aiming once perturbations exceed ~10°. And, prior work also shows that participants often struggle to eliminate strategic adjustments during exclusion trials. Thus, the apparent "implicit" deviation may reflect persistent explicit strategy use rather than an implicit selection bias.

We agree that this is an important issue to consider. However, there are a number of results in the current study that make it unlikely that “switching” difficulty of explicit strategies is the source of implicit aiming.

First, to ensure participants fully understood the critical distinction between "move directly to the target" and "make the cursor hit the target," we included two instructed practice blocks. In these, a 70° rotation was presented for a short block, followed by a trial in which the perturbation was eliminated, with participants instructed to adopt an aiming strategy when the perturbation was present and stop aiming when it was removed. Importantly, participants were able to revert back to baseline behavior within a single trial, demonstrating that they both 1) understood the instructions and 2) were fully capable of switching off an adopted explicit strategy. We employed the same instructions following the gradual rotation blocks. Yet here, the participants showed a large aftereffect when the perturbation was turned off.

Second, and perhaps most compelling, we show that simply changing the cover story in a way that biases participants' attribution of the perturbation source has a marked change (5-fold) on the size of the aftereffect. It is difficult to reconcile this with the hypothesis that implicit aiming is really the residual effect of failing to terminate an aiming strategy. In contrast, the cover story effect is consistent with the idea that participants’ inference about the source of the error has been changed, thereby influencing how much of the learned compensation is retained.

Third, we are unaware of any empirical result that has formally demonstrated that participants have difficulty disengaging explicit strategies. To our knowledge, this concern has been raised only as a discussion point in some papers. Importantly, those studies (e.g., 't Hart & Henriques, 2025; Chen & Taylor, 2026) used designs in which participants alternated between include-strategy and exclude-strategy trials on a trial-by-trial basis. These designs at, a minimum, require participants to carefully follow instructions (Chen & Taylor 2026) and could impose a non-trivial cognitive switching demand, which could plausibly produce strategy carry-over. By contrast, our experiment used a block design, in which participants were instructed to disengage their strategy for the entire duration of the aftereffect block. Given that a substantial body of work indicates a cost when maintaining an explicit strategy (Haith et al., 2015; McDougle & Taylor, 2019; Kim, Velazquez-Vargas, & Taylor, 2024), we consider it unlikely that the substantial aftereffects observed at the end of aftereffect block were due to the continued use of a (residual) explicit strategy. In addition, it is unlikely given the short duration of the study that participants developed a response-selection habit that would prove difficult to suppress – an effect that takes thousands of trials to develop and emerges only under time-pressure (Du & Haith, 2025; Hardwick et al., 2019).

Taken together, we do not think the aftereffects observed here can be parsimoniously attributed to a limited ability to suppress the use of an explicit strategy. We have added text concerning this alternative hypothesis in the Discussion (Lines 489-500):

*“One alternative interpretation of the aftereffects observed in Exp 1-2 is that they reflect a limited ability to disengage an explicit aiming strategy rather than a distinct implicit aiming process(’t Hart et al., 2024). However, several features of our data argue against this account. First, in the instructed practice blocks preceding the main experiment, participants reverted to baseline behavior within a single trial when instructed to stop aiming following an abrupt 70° rotation, demonstrating that they both understood the instructions and could fully switch off an explicit strategy. Second, manipulating the cover story alone strongly modulates the aftereffect magnitude, a result that is difficult to reconcile with a strategy-disengagement account but follows naturally from a credit assignment framework. Third, explicit aiming is cognitively costly (Haith et al., 2015, McDougle & Taylor 2019; Kim et al., 2024), making it unlikely that participants would continue to maintain a residual strategy throughout the aftereffect block in the absence of any task benefit. Finally, participants are unlikely to have developed a response-selection “habit” that would persist given that such habits typically require many more trials than experienced in our study (Hardwick et al., 2019; Du et al., 2022).”*

(b) Instructional effects do not uniquely support a credit-assignment mechanism
The authors argue that priming participants to attribute perturbations externally or internally modulates implicit aiming, ruling out a transition from explicit to implicit processes. Yet instruction manipulations are well known to influence explicit re-aiming patterns. For instance, external priming typically increases strategic re-aiming, which can reduce the residual error available to appear as "implicit" during probe trials. Conversely, internal priming would reduce explicit compensation, increasing the residual. Thus, these findings are consistent with the hypothesis that implicit aiming reflects incomplete suppression of explicit strategies.

We agree with the reviewer that instructions can influence explicit re-aiming; indeed, we exploit this in Exp 2. However, our data argues against the view that the residual "implicit aiming" reflects incomplete suppression of explicit strategies. First, total learning during training was equivalent across the three conditions (now quantified in Fig. S2). If the instructions modulated the explicit component, as the reviewer's account requires, then equivalent total learning implies that a separate process must have compensated for the differing explicit contributions. This compensatory process cannot be implicit recalibration, because the delayed feedback used here would have largely suppressed it. Some additional implicit process is therefore required. In other words, the very conditions the reviewer invokes to explain the residual error end up mandating the existence of implicit aiming.

Second, the reviewer's hypothesis makes a directional prediction that our data contradicts. Superficially, if the “implicit aiming” we observed reflects incomplete suppression of explicit strategies, one would anticipate a positive correlation between aftereffect and the magnitude of the explicit strategy. In contrast, following our model, explicit aiming and implicit aiming complement each other and should be negatively correlated. The "priming explicit" condition, in which participants were fully informed of the perturbation schedule, should engage explicit processes more strongly than the uninstructed group in Exp 1. If implicit aiming were merely the incomplete suppression of an explicit strategy, one would predict a larger aftereffect in the priming-explicit condition than in Exp 1. We observed the opposite: the priming-explicit condition produced the smallest aftereffect, consistent with our complementary model.

In fact, if causal attribution is central to this putative implicit component, perturbation size would provide a more direct manipulation, as it is known to reliably shift attribution between internal and external causes. I would suggest the authors to use this existing technique to test the hypothesis of causal attribution.

We agree that varying perturbation size would provide complimentary evidence. However, we thought that varying the instructions was a more direct way to manipulate credit assignment since the manipulation is directly targeting the attribution process. That is, we are priming/biasing how they will solve the credit assignment problem while, importantly, keeping everything else the same in the experiment. Varying the size of the perturbation seems less direct in that the participant must infer the likely source of the error.

That said, we agree that manipulating perturbation size provides another probe of implicit aiming. Although not the main focus of the experiment, variation in perturbation size was baked into Exp 1 in that we administered probes at multiple time points during the gradual rotation phase. We found that the magnitude of the aftereffect was relatively constant across these three probes (P1: 10.0 ± 2.1°, P2: 13.0 ± 3.7°; P3: 13.6 ± 2.1°). The near-constant aftereffect across these probes is consistent with our credit assignment account: the motor system appears to attribute at most ~10° of error to internal motor bias (Wei & Kording, 2008, Kim, Chua, Hu, 2025), with any remaining error attributed to the external perturbation.

We did not report the perturbation size analysis in the original manuscript. We now include the following in the revised Results (Lines 204-207):

*“Consistent with the credit assignment account, the aftereffects were all around 10–15° across substantial differences in rotation size and training duration at the time of the three probes, with any remaining error attributed to the external perturbation. This range is similar to the maximal magnitude of error that participants typically attribute to internal motor biases*(Kim et al., 2025; Wei & Körding, 2009)*”*

3. Interpretation of Experiment 3 and alternative accounts
Experiment 3 reports that implicit learning persists under limited reaction time, whereas explicit re-aiming does not, and that implicit aiming exhibits savings. This is presented as a dissociation between explicit and implicit aiming and a dissociation between implicit aiming and implicit recalibration (the latter has no savings). 
However, limited preparation time introduces incentives for participants to initiate movements rapidly, possibly limiting the expression of explicit re-aiming but not necessarily eliminating it. Residual strategic adjustments may manifest as the observed "implicit aiming" effect. Moreover, the observed learning resembles (in size and temporal profile) results from RT-limited tasks without delayed feedback (Albert et al., 2022), suggesting that the learning in Fig. 3D does not depend specifically on delayed feedback. It is also unclear whether the implicit components inferred in Experiments 1-2 (relying on delayed feedback) and Experiment 3 represent the same process.

While it is possible that the implicit effects observed in Exps 1 and 2 are different from those observed in Exp 3, we employed a different task in Exp 3 with one goal being to obtain converging evidence of implicit aiming. However, we used the same method to probe implicit aiming: an aftereffect block in which we instructed the participants to abandon the strategies and move directly towards the target. The aftereffect was very similar in magnitude to the hand angle at the end of learning (Learning: d=1.97°, ts(12)=1.7, p=0.11; Relearning: d=1.99°, ts(12)=1.9, p=0.08). We take this to indicate learning here is mostly implicit (our intended effect). There was a ~2° drop in the aftereffect which could reflect an explicit strategy, However, even if there was some small contribution of explicit aiming, it would not account for the observed savings effect of ~10°.

Upon revisit our manuscript, we notice that this critical point was missing in the original submission. We have added the following sentences when discussing the results of Exp 3 (Lines 292-294):

*“We included probe trials after the training block, asking participants to move direct to the target. We observed similar hand angle on these probe trials compared to late learning (ds<2.0°, ts(12)<1.9, ps>0.08), suggesting that learning was mostly implicit.”*

More generally, we think it is problematic to directly compare learning functions between our study and Albert et al. (2022) given the differences in methods and experimental design. The absolute magnitude of learning is dependent on many factors. For example, while delaying feedback attenuates implicit recalibration, it typically results in even faster learning compared to tasks using on-line feedback, a task in which learning involves both implicit and explicit contributions (Albert et al., 2021 NHB; 2022 eLife). This indicates that learning processes operate quite differently in isolation than in combination. Indeed, a core claim of Albert et al. (2022) is that when the two processes operate together, the contribution of each is less than that observed when studied in relative isolation, an effect they attribute to the two processes competing for a common error signal.

Finally, we note that the aftereffects and rate of forgetting were comparable across Exp 1-3, providing converging evidence of a shared implicit (aiming) process in these experiments. Please see our response to the last comment of Reviewer 2 for details.

Furthermore, savings in Experiment 3 does not uniquely support the existence of implicit aiming. The empirical patterns replicate known findings: RT constraints preserve savings, and error clamps do not lead to savings. The interpretation relies on the assumption that delayed feedback isolates implicit aiming and clamp feedback isolates implicit recalibration—an assumption that should be tested (specifically for implicit aiming) rather than taken as given.

The assumptions underlying Exp 3 are well supported by the literature. Delayed feedback suppresses (or even eliminates implicit recalibration (Brudner et al., 2016; Schween et al., 2017; Wang et al., 2024; Hadjiosif et al., 2023) and error clamps discourage explicit strategy use (Morehead et al., 2017; Kim et al., 2018, 2019; Tsay et al., 2022). Our control experiment using delayed feedback + clamp show no adaptation, suggesting no implicit (or explicit) aiming operation under clamp feedback. As such, we believe it is well-supported that clamp feedback isolates implicit recalibration. We isolated explicit aiming with delayed feedback + limited RT. Given the evidence that delayed feedback eliminates implicit recalibration, the remaining question is whether limited RT sufficiently suppresses explicit strategy use. As noted in our proceeding response, the aftereffect was comparable to late learning, consistent with the idea that learning is implicit. In sum, we believe the empirical evidence strongly validates our manipulations.

The implicit aiming also has a magnitude issue. If Experiment 3 validly isolates implicit aiming (limited RT + delayed feedback) and implicit recalibration (limited RT + clamps), then both components independently reach ~20°. Their sum (~40°) far exceeds typical adaptation magnitudes observed in visuomotor rotation studies. This suggests that either the theoretical framework in Figure 1 is flawed (implicit aiming and implicit recalibration are independent and additive, along with other components), or the magnitude estimation—or the underlying assumptions—are problematic.

Again, we think summing learning curves across experimental approaches is problematic. In experiments that use manipulations to isolate components, each process itself must compensate (if possible) for the perturbation (e.g., a 30° rotation). In experiments in which multiple processes operation, they combine to compensate for the perturbation, with each process relying, more or less, on different error signals (e.g., sensory-prediction errors, task performance errors, etc.) to yield total adaptation. For example, in a standard visuomotor rotation experiment with a large perturbation, say 45°, implicit recalibration may absorb ~20° of error based on sensory-prediction errors while re-aiming strategies may compensate for 25° of task-performance error (and this could be a combination of 15° for explicit and 10° of implicit re-aiming). Once manipulations are imposed to suppress the operation of one or more of these processes, these ratios would necessarily change. Complicating matters, these ratios could change over time as the error signals themselves are changing. For example, under a limited RT + clamp condition, the error does not diminish over the course of “learning” whereas it does when the feedback is contingent on hand position (as in the limited RT + delayed feedback condition). As a result, attempting to simply add them from “isolation” paradigms is likely to be ill-posed; indeed, attempts to do this have yielded mixed findings (Maresch and Donchin 2020; t’Hart et al., 2024; Chen & Taylor 2026; Liddy 2026). For these reasons, we do not believe that one can generate a useful reference point by simply summing the learning curves of delayed feedback and clamp experiments together.

4. Interpretation of Experiment 4 and the role of explicit processes
Experiment 4 begins with a 30° abrupt rotation under delayed feedback, which almost certainly engages explicit re-aiming. As the perturbation gradually decreases, participants may become less aware of their re-aiming and shift toward habitual or automated behavior. The observed "implicit aiming" may thus reflect a transition from explicit to implicit re-aiming rather than evidence against such a transition. This interpretation conflicts with the manuscript's repeated claim that implicit aiming is not derived from explicit learning.

To be clear, the specific claim we made in the manuscript is that implicit aiming is unlikely to be driven by the habitualization of an explicit aiming strategy. As noted above, we consider implicit and explicit aiming to share a common inference process. As such, one component can be derived from the other in a conditional manner: attributing a portion of the error to the implicit component leaves the residual to be assigned to the explicit component, and vice versa.

Regarding the 30° abrupt rotation manipulation, we would like to highlight three points:

First, a critical feature of Exp 4 is that we applied the 30° perturbation with no preceding baseline block of veridical feedback. A typical adaptation experiment begins with a veridical-feedback baseline block, allowing participants to build a clear prior about their own motor bias and noise; consequently, when they suddenly encounter a 30° shift, they can be fairly certain that something has changed in the environment. In contrast, participants in Exp. 4 had no opportunity to sample their true bias and variance, and they never experienced an abrupt change in feedback. We therefore reasoned that they might attribute most of the 30° perturbation to an internal source rather than to an external perturbation. Consistent with this assumption, the aftereffect was comparable in magnitude to late learning, suggesting that adaptation was largely implicit.

Second, 30° is a tricky sized perturbation. While it exceeds motor or perceptual noise, and thus, participants should detect that the feedback has been perturbed, the operation of implicit adaptation can quickly obscure the picture. Indeed, in the Hadjiosif, Morehead, and Smith (2023) study (the design we mirrored), minimal explicit learning was observed with an abrupt 30° rotation. In our design, implicit recalibration was suppressed by delayed feedback, but we mimicked its impact on performance by ramping down the rotation, such that the maximum perturbation participants compensated for was approximately 20°. Given the convergent results of our Exp 4 and Hadjiosif, Morehead, and Smith (2023), it seems to be fair to claim the learning in this design is largely implicit (also see point three below).

Third, we acknowledge that, based on the aftereffect alone, we cannot rule out the possibility that an explicit component was present early in training. We conducted a control condition in Experiment 4 in which a protracted feedback baseline block preceded a 30° rotation. In this condition, a clear explicit component emerged, and the aftereffect dropped by approximately 50% — a pattern more consistent with what we understand the reviewer to have in mind from a "typical" design. This result suggests that if explicit adaptation is indeed involved in learning, it is likely to be reflected in the aftereffect, regardless of the gradual rotation. Taking the most skeptical view, it remains possible that a small explicit component was present early in Exp. 4 and was fully suppressed before the aftereffect block. Under our credit-assignment model, the system can reattribute the error as the gradual rotation unfolds, increasing the inferred bias while decreasing the inferred perturbation. Even this possibility, however, is broadly compatible with our framework rather than standing against it.

Taken together, we do not think the results of Exp 4 provide substantial evidence that explicit aiming transitions into implicit aiming. Moreover, the credit-assignment hypothesis provides a parsimonious account regardless of whether such a transition occurs, offering a coherent and consistent interpretation across all experiments.

Per the reviewer’s comment, we revised the paragraph when we introduce the methods of Exp 4 and how the manipulation of gradual rotation and no veridical feedback may bias the learning here to be implicit (Lines 316-322):

*“First, we initially introduced a 30° rotation and then gradually decreased the rotation to 15° over 100 trials. This gradual reduction was included to mimic how the error signal diminishes in classic visuomotor rotation tasks due to implicit recalibration. Second, we did not provide any feedback during the baseline phase, simply introducing the 30° rotation on the first trial with cursor feedback. We reasoned that, in the absence of veridical feedback, participants would be more likely to attribute the resulting error to their own motor bias rather than to an external perturbation, and as such, learning would be more likely to be implicit.”*

Lines 341-344:

*“The average heading angle in the aftereffect block was very similar to late perturbation performance (Fig 4A, t(29) = 0.8, p=0.45), suggesting that learning here was almost all due to implicit aiming. This observation is consistent with what has been observed in Hadjiosif et al, the design we mimicked in Exp 4.”*

By the way, if implicit aiming is governed by contextual inference, the COIN model should also be fit to Experiments 1-2, where implicit aiming coexists with other forms of learning. Such model fits would be essential to support the theoretical claim about the origin of implicit aiming.

This comment had special meaning for us given an extended correspondence we had with Máté Lengyel and Daniel Wolpert on COIN. Lengyel and Wolpert made the point that the COIN model in its current form is limited in how it accounts for learning in response to a gradual rotation. Specifically, the model tends to treat the current context as the baseline context even as the perturbation grows large. A paradoxical prediction of the model (and one not supported by data) is that learning under a gradual rotation will result in a stronger aiming strategy compared to an abrupt rotation. Diagnosing the source of this paradoxical prediction would require substantial model development, beyond the scope of the present work and tangential to our core findings.

5. Conceptual framing and theoretical contribution
The authors propose replacing the explicit-implicit taxonomy with an action-selection vs. action-execution taxonomy. While this perspective is valuable and consistent with prior discussions in the field (e.g., Krakauer 2019), the new framework does not eliminate the need to differentiate explicit and implicit processes; rather, it introduces a new explicit/implicit division within the action-selection branch. The manuscript does not fully resolve how the two putative action-selection processes—explicit and implicit aiming—can be behaviorally distinguished, nor how future studies should reliably elicit and measure implicit aiming.

We respectfully disagree with this assessment. While we recognize that the selection/execution distinction has been discussed in some papers, the adaptation literature has focused on explicit/implicit as the primary branch. As such, we see value in our revamped hierarchy of processes for sensorimotor adaptation. By doing so, we highlight that explicit and implicit aiming share similar computational properties (e.g., showing savings rather than attenuation; being sensitive to contexts) that are clearly distinct from implicit recalibration. We argue that developing computational models for sensorimotor adaptation at the level of action selection and execution can offer greater explanatory power than the implicit/explicit dichotomy. This reframing is directly motivated by the debate over apparently contradictory results concerning the properties of the implicit system, including differences in temporal dynamics and contextual sensitivity. We believe our framework provides a principled basis for reconciling these conflicting findings and integrating them into a more cohesive theory of sensorimotor adaptation.

We also wish to emphasize that the implicit/explicit distinction in sensorimotor adaptation has always been defined at the phenomenological level (Taylor et al., 2014; Taylor & Ivry, 2014). In the current study, we use multiple experimental designs to demonstrate the existence of an implicit process that operates in the absence of implicit recalibration, evidenced by residual aftereffects in conditions where participants subjectively believe they are moving directly toward the target. We have further shown that implicit aiming can be selectively introduced or isolated using gradual rotation and limited RT combined with delayed feedback. Crucially, we have proposed a mechanistic account of the origins of implicit aiming, rooted in the credit assignment process between external and internal sources of error. This account provides a coherent interpretation across the set of experiments.

In summary, we believe the present work not only offers a valuable framework that incorporates and extends current theories of motor learning, but also provides concrete empirical tools for further testing the properties and origins of implicit aiming.

Minor Comments
* Line 136: The manipulation check mentions a 65° rotation, while the figure legend indicates 70°.

Corrected-- it was 70°.

* Line 258: The assumption that implicit aiming is absent during clamp feedback because participants are instructed to aim directly at the target contradicts the central claim that implicit aiming occurs even when explicit aiming is prevented by instruction.

To be clear, we do not characterize implicit aiming as an automatic process. As the priming manipulations in Exp 2 demonstrate, it is modulated by instructions and is goal-relevant, unlike implicit recalibration, which operates automatically.

We don’t follow the reviewer’s claim of a contradiction. We assume implicit aiming persists into the aftereffect because the system should not remove its own internal bias whether external perturbation exits or not. By contrast, the component compensating for an external perturbation should be disengaged when the perturbation is no longer present.

* Figure 4: The interpretation of the drop as "implicit" is unclear. A 30° abrupt perturbation typically elicits explicit responses; attributing the drop solely to implicit processes requires stronger justification.

Please see our response to your comment #4 for details. In brief, we mirrored the training procedure of a previously reported work (Hadjiosif, Morehead, and Smith, 2023) where they have shown that learning/drop is largely implicit. Moreover, we applied a special design using no veridical feedback baseline, which is likely to bias the error observed at the beginning of the perturbed block to internal motor bias and as such increase the possibility that learning is implicit. Consistent with those assumptions, we observed almost no change in hand angle between late learning and aftereffect block, and this is not the case in the control condition where we applied prolonged baseline block with veridical feedback. Putting things together, we believe there is minimal explicit aiming in this condition.

* Line 385: The explanation for anti-savings in the CPC model relies on interference from opposite-direction errors, yet anti-savings has been observed even without such exposure.

We recognize that there are conflicting perspectives on this issue. Nonetheless, we think the core observations are consistent with the CPC model. The literature indicates that anti-savings is only observed when participants receive feedback during a washout phase (or during a baseline phase). While manipulations have been used to minimize the experienced error, there is still some error in the opposite direction when feedback is available (Avraham & Ivry, 2025; Avraham et al., 2021). Moreover, as shown previously, even a one-degree clamp is sufficient to drive substantial adaptation (Kim et al., 2018). In our CPC model (Wang and Ivry, 2025), these small opposing errors are sufficient to build up learning in opposing units, thereby producing the observed attenuation. By contrast, washout without feedback produces no such attenuation (Avraham & Ivry, 2025).

* The rationale for mimicking the reduction of error signals in Experiment 4 is insufficiently explained. The manuscript has not clearly articulated how these error dynamics relate to implicit aiming.

As mentioned above, in the original design of Hadjiosif et al. (2023), movement-synchronized feedback was applied under limited reaction time conditions such that both implicit aiming and implicit recalibration could contribute to adaptation. Consequently, although the applied perturbation was constant in size, the error signal experienced by the implicit aiming system was not constant. It decreased gradually over time as implicit recalibration progressively offset the perturbation. To account for this in our replication attempt, we estimated the "effective" error size driving implicit aiming by first simulating implicit recalibration using a state-space model, then subtracting its trial-by-trial contribution from the applied perturbation.

We have now clarified this procedure in both Results (see our response to your comment 4) and Methods section (Lines 730-736):

*“The rotation size was initially set to 30° and then gradually decreased to 15° over the course of the block following an exponential decay. This perturbation schedule was designed to approximate the time course of errors that were experienced with a visuomotor rotation task in Hadjiosif et al. Part of the error in the classic visuomotor rotation task is compensated by implicit recalibration; thus, the error signal relevant for the aiming system decreased across time. Specifically, the contribution of the hypothetical implicit recalibration process was simulated using a state space model with a learning rate of 0.05 and retention rate of 0.95.”*

* Figure 5E: Labels for "learning" and "relearning" appear incorrect.

Corrected

**Reviewer #2:** This manuscript presents a series of four primary experiments that try to establish implicit selection/aiming and implicit calibration as functionally separate motor learning processes--both dissociable form explicit action selection. The experiments are designed in a thoughtful and meticulous manner and appear to show a clear dissociation between aiming and calibration with in part, opposite patterns of aftereffects and savings. These results suggest that it may be more useful and parsimonious to recast motor learning processes in terms of a functional/computational division between action selection versus execution rather than in terms of the traditional implicit/explicit division. Importantly, implicit selection and calibration are also explicitly tied to two different, existing computational models.

Thanks for the nice summary of our work!

I have one general, mostly expository issue and one specific question:

The fact that the series of experiments provides a tour de force through key manipulations used in sensorimotor adaptation paradigms (e.g., clamped feedback, feedback delays, savings, small versus large perturbations, transparent versus non-transparent perturbations, explicit framing) is a clear strength, but also a potential challenge for readers not fully immersed in this literature (which includes this reviewer). There are a lot of individual experimental details and assumptions about the effects of specific manipulations that need to be absorbed/integrated to grasp the whole story. While this cannot be avoided completely, I believe that more could be done to improve accessibility for a broad readership. Specifically, it would be helpful to show an advanced organizer (as graphic or table) of the core elements of the paradigm, the individual experiments, and the role of the various manipulations.

We appreciate the recommendation to make the paper more accessible. We have added a new panel to Figure 1, the Venn diagram shown below. Here we depict how widely used methods for studying adaptation map onto learning constructs, comparing a classic framework and our new framework. Of note here, in the classic framework, limiting RT and instructing participants to aim to target are assumed to exclude explicit learning and thus, by default probe a singular implicit process (or undefined set of implicit processes). In our framework, these experimental manipulations can contribute to implicit recalibration or implicit aiming. By the classic view, combining delayed feedback with either limited RT or "aim-to-target" would result in no learning; our model suggests that implicit aiming would operate under these combinations. We test this idea combining "aim-to-target" with delayed feedback in Exp 1 and combined limited RT with delayed feedback in Exp 3. We added this diagram so that the reader can graphically see how the design of the experiments is motivated by the theoretical framework.

We also revised the last paragraph of the Introduction to preview our methodological choices, framed by this Venn diagram (Lines 89-96):

*“In the present study, we conducted a series of experiments to explore implicit contributions to action selection, a process we refer to as implicit aiming. We employed a variety of methods to identify and characterize this process, highlighting how it is distinct from implicit recalibration (Fig 1C). In particular, we combined delayed feedback, a method that excludes implicit recalibration, with manipulations designed to exclude explicit aiming such as instructing participants to stop using an aiming strategy and reach directly to the target or limiting reaction time. Both designs revealed prominent learning, providing evidence for the existence of implicit aiming. Moreover, we show that implicit aiming and implicit recalibration differ in their temporal dynamics and sensitivity to contextual information.”*

My specific question (that may reflect my specific difficulty with integrating information across the diverse set of experiments) regards the difference between Experiments 1/2 versus 3. If I understand correctly, the implicit aiming bias results from misattributing perturbations to internal factors, which requires a situation with non-transparent perturbations (i.e., the gradual changes in Experiments 1 and 2). Yet, perturbations in Experiment 3 were very obvious and similar to the ones used in the control condition of Experiment 1 that bias-free re-aiming. How then can the authors be sure that the re-learning effects seen in that experiment reflect the same process implicit bias that was observed in Experiments 1/2?

There are several issues we need to clarify here and in the main text:

First, a gradual rotation is not a prerequisite for engaging implicit aiming. To measure implicit aiming, we employed methods that discourage explicit aiming and implicit recalibration, allowing us to probe for behavioral signatures of implicit aiming. One such method is to introduce the perturbation in a gradual manner. When the perturbation gets large, participants will use an aiming strategy (Modchalingam et al, 2023., which is why we didn’t include this method in the Venn diagram). But there is much more uncertainly than with an abrupt perturbation. Most important, explicit aiming should be aborted when participants are told to aim to the target” prior to the aftereffect block in Exp 1-2. Limiting RT is a relatively artificial method, but based on prior work, appears to greatly attenuate the use of an explicit aiming strategy (Albert et al, 2021; 2022; Leow et al., 2017).

Moreover, we hypothesis that implicit aiming arises from a credit assignment process, namely when an observed error is (mis)attributed to some internal state variable such as a motor bias or proprioceptive drift. Introducing the perturbation in a gradual manner (Exps 1 and 2) with delayed feedback is one way to boost implicit aiming as the incremental nature of the error may bias credit assignment toward internal sources. Limiting reaction time (Exp 3) with delayed feedback is another way to boost implicit aiming, presumably because the increase in baseline motor error biases the system to interpret movement errors as internally generated. We also note that it was a deliberate choice to use different methods in Exps 1 and 3, seeking converging evidence of implicit aiming.

Upon reading our Results again, we realize that the gradual rotation was introduced in parallel with the delayed feedback, making it sounds like a method to exclude explicit aiming. We have revised this section to avoid this confusion (lines 121-128).

*“To probe for the presence of an implicit process associated with action selection, we delayed the endpoint feedback by 1.5 s, a manipulation that has been shown to essentially abolish implicit recalibration*(Brudner et al., 2016; Schween & Hegele, 2017)*,*(Samuel D. McDougle & Taylor, 2019)*. To disrupt explicit aiming, we introduced the perturbation in a gradual manner, increasing the angular perturbation by 0.5°/trial up to a maximum value of 70°. While participants would become aware that the visual feedback is perturbed at a certain point, we assume that the uncertainty introduced by the gradual change limits the utility of a specific aiming strategy*(Holland et al., 2018; Kagerer et al., 1997; van Mastrigt et al., 2023)*. To further increase the uncertainty of the perturbation size we gradually decreased the rotation from 70° to 25° over the second half of training. To assess the contribution of an implicit component to the observed changes in behavior, we inserted probe blocks (P1-P3) in which no visual feedback was provided, and participants were instructed to move directly to the target* (Maresch et al., 2021; Taylor et al., 2014) *(Fig. 2A). We attribute systematic deviations in reaching direction on these trials to implicit aiming.”*

Second, the reviewer noted that the instructed block in Exps 1 and 3 involves an abrupt rotation. However, there are several important differences. One is the limited RT manipulation describe above. Another is the size of the perturbation. In Exp 1, the perturbation rose up to 70°, a value that would unlikely to be attributed to an internal bias. In contrast, the perturbation in Experiment 3 was much smaller, peaking at 30°.

Third, we acknowledge that we cannot be certain that the exact same implicit aiming process is operative in Exps 1–2 and Exp 3. Beyond an argument from parsimony, we note that Exps 2–4 were designed to test a priori predictions we derived after observing the aftereffects in Exp 1, the initial result in this project that suggested a novel implicit learning process. The learning observed in Exps 1 and 3 shares several notable similarities, suggesting that the same process is at work despite the differences in how the perturbation was introduced. (a) The magnitude of learning was comparable between the aftereffects in Exps 1 and 3 (10~13°). (b) The forgetting rate was also similar across Exps 1 and 3 (~0.01, see figure on the left), and this value was substantially lower than that of explicit aiming typically observed in sensorimotor adaptation tasks (>0.1; Smith et al., 2006; McDougle et al., 2015). We note that this low forgetting rate is consistent with our credit assignment account, since internal biases are, by nature, assumed to be temporally stable and therefore not readily discarded.

We hope the Venn diagram above along with the revisions to the text clarifies the motivation for applying different methods to isolate implicit aiming. Per the reviewer's suggestion, we have also added the following in the Discussion (Lines 450-463).

*“A central goal of the present study was to isolate implicit aiming by minimizing or eliminating the contribution of implicit recalibration and explicit aiming. We combined delayed feedback (which excludes implicit recalibration) with two methods for excluding explicit aiming, instructing participants to aim directly to the target*(Hadjiosif et al., 2023; Taylor et al., 2014) *(Exps 1–4) and limiting reaction time during abrupt perturbation training*(Albert et al., 2022; Haith et al., 2016) *(Exp 3). Although these experiments employed substantially different training schedules, we observed a prominent implicit component across the experiments with striking quantitative similarities. First, the magnitude of the implicit aiming component was comparable in Exps 1 and 3 (10–13°) under conditions in which credit assignment was not biased by instructions. Second, we observed a consistently low forgetting rate across the experiments (~0.01, Fig S6). This value is substantially lower than the forgetting rate typically observed when explicit aiming is involved (>0.1)(McDougle et al., 2015; Smith et al., 2006), providing additional evidence that implicit aiming is not a residual component of explicit aiming. More important, the slow rate is in accord with the credit assignment account of implicit aiming. Internal biases are, by nature, assumed to be temporally stable. Given this, a corrective process to offset the bias (i.e., implicit aiming) should also be stable(Forano & Franklin, 2020; Kim et al., 2015).”*

**Reviewer #3:** In this interesting paper, the authors aim to demonstrate the existence of two forms of implicit processes in visuo-motor adaptation. An implicit "aiming" action selection process and an implicit "recalibration" action execution process. The results of Figure 3 (which, according to the authors themselves, are the most convincing, and I agree) are particularly interesting, as they show a dissociation of these processes in savings, with recalibration showing (known) anti-savings and aiming showing savings.

Thanks for these summary comments.

Experiment S2 is also well-designed and interesting in its own right, and could be promoted to the main part of the paper.

Exp S2 was designed to confirm that the aftereffect observed in Exp 1 is not driven by use-dependent learning. We refer to it in the main text as a control experiment (Line 157-159). After giving some thought to the Reviewer’s recommendation, we have opted to keep it in the Supplementary Materials for two reasons. First, a key comparison in the paper is between Exp 1 and Exp 2, which together demonstrate the effect of instruction on implicit aiming. Inserting Exp S2 between them would interrupt this flow. Second, the Results section is quite long and the manuscript is close to the Journal's length limit. Moreover, the design of Exp S2 requires some additional explanation that seems better suited to the Supplementary Materials. That said, we would be happy to move it into the main text if the reviewer and editor believe this would be best for the paper.

Major: 

Since the results of the forced response time task in Experiment 3 are the most critical, extreme care should be taken in both the experimental design and the description of the results. A major concern is the very short washout of 30 trials; visual inspection suggests that the washout was incomplete, as the hand direction in the first cycle of the relearning block does not start at 0. At the very least, the washout data should be presented. Preferably, a control experiment with a longer washout should be performed.

We used a short washout in the original Exp 3 based on pilot work showing that this was sufficient to bring behavior back to baseline. Note that we erroneously reported that this was a 30-trial washout when it was actually 60 trials. However, as noted by the reviewer, it turned out that we did succeed in bringing behavior back to baseline in the actual experiment with a larger sample.

Following the suggestion of the reviewer, we have now run a new limited RT experiment (n=13) with a longer washout phase of 90 trials (see figure below). Hand angle at the end of washout was back to baseline (t(12) = 0.15, p = 0.88, see figure on the right). Importantly, we replicate the dissociation between implicit aiming and implicit recalibration observed in the original experiment (Fig 3, see below, Panels D and F). Relearning was much faster than original learning when we delayed the feedback and slower when we used clamped feedback (that wasn’t delayed).

While we could add this experiment as a replication of Exp 3, we opted to replace the original Exp 3 with this new experiment in the revision since the washout was complete. In addition, we now include the entire washout block in the new Figure 3.

**Fig 3**

Related, multiple details are missing. What is the rotation imposed in the washout block? What is a "Cycle"? There are three targets; how is hand direction measured across them? What is "10d"?

Thank you for bringing this to our attention. Veridical feedback (no rotation) was provided during the washout. Each cycle is composed of 3 trials. Note that we randomized the target sequence so that each target appeared twice every six trials. For this reason, the learning curve is plotted with a resolution of two cycles. The error is measured as the difference between the target position and the hand reaching angle when the amplitude of the hand movement reaches the target distance. 10d is 10°.

We have revised the manuscript to include this information (Lines 294) and are now consistent in our use of the degree symbol.

Unfortunately, Experiment 1 (and similarly Experiment 2) is relatively weak with several issues. First, the results rely on participants following confusing instructions. In the rotation, participants were instructed to "hit the target with the cursor". Then, in the rotation block, they "were instructed to move directly to the target" (!).  The lack of feedback may contribute to the fast decay, but it is unclear how these instructions work. The control experiment in the inset is not really convincing either, since it uses an abrupt perturbation and participants are instructed about it. In contrast, the gradual rotation in the main experiment is introduced to limit awareness. But then, because the gradual rotation is so large (unclear rationale for this choice), the authors still admit that the participants do become aware of the rotation. This is all somewhat confusing.

There are several points we need to clarify:

First, we recognize that these instructions can be confusing—something that has been discussed in other papers and we’ve carefully considered in prior studies from our lab using similar manipulations. This concern was the main reason we decided to include two demonstration blocks in the current study. Specifically, we included two abrupt 70*°* rotation blocks in which the participants were informed with the perturbation and were asked to “*make the cursor hit the target*”. Here participants readily re-aimed to compensate for the rotation. When we removed the perturbation and instructed the participants to “*reach directly to the target*”, they were also able to return to baseline, usually within a single trial. We repeated this procedure twice to really hammer home the difference in instructions and, based on the results, are confident that the participants were able to flexibly switch between the two instructions at least before the formal experiment. Importantly, we provided the same set of instructions before the onset of the gradual rotation block and before the start of the aftereffect block. While the perturbation was gradual here, it seems reasonable to assume that participants recognized the need to re-aim as the error got larger, and correspondingly, were able to abandon their aiming strategy during the aftereffect block. We do believe that relative to prior studies that have involved similar manipulations of the instructions, our new method represents a significant improvement to make these instructions as clear as possible.

Second, while there was some residual error in the aftereffect block—what we attribute to implicit aiming, there was also a large change in heading angle on the first trial of the aftereffect block, providing evidence that the participants did follow the instruction to abandon the strategy. We don’t think this single-trial drop could be explained by forgetting as it is qualitatively different from the change in hand angle between the second and the first trial in the aftereffect block (See figure below). Notably, we assume the residual error arises because of a credit assignment problem. Namely, they misattribute some of the observed error during the rotation phase to internal bias. This bias would persist in the aftereffect block (given there is no feedback) since the participants believe that offsetting their internal bias it is essential for accurate reaching.

Third, we acknowledge that there are potential limitations in probing implicit processes with either "aim-to-target" or limited reaction time. Nevertheless, these are the two principal methods widely used in the field (e.g., Hadjiosif, Morehead, and Smith, 2023; McDougle and Taylor, 2019). In particular, the "aim-to-target" instruction was used in the original work establishing the implicit/explicit distinction in sensorimotor adaptation (Taylor, Krakauer, Ivry, 2014). We also note, while people have suggested that the "aim-to-target" instruction or limiting reaction time are not the cleanest way to measure implicit recalibration, the current work, to our knowledge, is the first to provide a formal explanation and systematic test why this might be case. We show that both methods, when combined with delayed feedback to suppress implicit recalibration, converged in revealing what we take to be the operation of a novel implicit aiming process. As such, the convergence suggests that implicit aiming is not an artifact of any specific design; rather, it appears to reflect a general computation broadly involved in sensorimotor adaptation.

Taken together, we believe participants had a clear understanding of when to apply and when to remove their explicit strategy, and that the observed aftereffects reflect ambiguity in credit assignment during the learning process rather than misunderstanding of the task instructions. We have substantially expanded the Results and Methods sections to more clearly describe our design and the steps taken to ensure that participants understood the instructions. We also now include the full instructions for Exps 1-2 as Appendix 1.

In the Results section, we have (Lines 137-145):

*“To ensure that the participants understood the instructions and could abandon explicit strategies on probe trials, we included two demonstration blocks before the main phase of the experiment. In each of these, we briefly introduced a 70° rotation for 20 trials. Participants were informed about the nature of the perturbation and instructed to apply strategies and to “make the cursor hit the target”. These short blocks were immediately followed by probe trials, asking participants to “move directly to the target”. While participants successfully adapted to the rotation, they showed minimal aftereffects (t(18)s<1.7; ps>0.1; Fig. 2A, intercept) in the subsequent probe trials, demonstrating that they were able to adopt and then suppress an aiming strategy. Given these results, we assume that the participants understood the instructions to stop using explicit aiming strategies on probe trials.”*

In the Method, we have (Lines 639-650):

*“To ensure that participants fully understand the instructions and could not only adopt an explicit strategy, but also flexibly alternate between using or abandoning that strategy, we included two demonstration blocks with a 70° rotation, one in the clockwise direction and one in the counterclockwise directions (20 trials per block). Participants were informed of the perturbation and instructed to re-aim in the opposite direction to “make the cursor hit the target”. Each rotation block was followed by a no-feedback block (15 trials, i.e., probe trials) and then a feedback block (10 trials). Importantly, before each no-feedback block, the participant was instructed to stop aiming and “move directly to the target”. Participants showed minimal aftereffects in these two no-feedback blocks indicating that they understood the instructions and were able to rapidly terminate their use of an explicit aiming strategy. The same set of instructions (“move directly to the target” vs “make the cursor hit the target” were applied in the formal experiment. A transcript of the instructions is provided in Appendix 1.”*

Minor:

There are multiple typos in the manuscript, including in figures. For instance, ExpS2 is called Exp 2 in Figure S1, etc.

Corrected

Analysis of the trial-by-trial decay in experiments 1 and 2 would be interesting.

Please see our response to your next comment.

Line 46 - yes, indeed, cite and in discussion, discuss studies showing ultra-slow processes (Forano and Franklin 2020; Kim, Ogawa et al. 2015); could one of the new uncovered implicit aiming processes be such an ultra-slow process since it stops decaying in Experiment 4? Please discuss.

Thanks for bringing this to our attention; we had not considered this issue. Following your suggestion, we analyzed the forgetting rate across the experiments. Specifically, we fit an exponential decay function at the group level and then ran a bootstrapping procedure 100 times. The measured distribution is relatively Gaussian (see figure below). Note that we allowed the retention rate to be >1, assuming such observations could be introduced by noise. Capping the retention at 1 would cause an artificial bimodal distribution. We analyzed all aftereffect blocks across Exp 1–4 with the only exception of P2 for Exp 1–2. The former all follow a perturbation of around the same size (20–30°) whereas P2 of Exp 1–2 followed a 70° rotation, making it less comparable to the other cases. We averaged the retention across aftereffect blocks within each experiment to reduce noise. The figure below shows the bootstrapped forgetting rate (1−retention) with mean (horizontal bar) and 95% CI (vertical bar).

“Figure S6. Bootstrapped forgetting rate for the aftereffect blocks of Exps 1–4. To estimate the distribution of retention values, we used a bootstrap procedure in which participants were resampled with replacement (100 iterations). On each iteration, the exponential decay function was refitted to the group-level data. Forgetting rates were then averaged across aftereffect blocks within each experiment. We did not include P2 of Exps 1–2 in this analysis given that the rotation size (70°) differs substantially from the other conditions (20–30°). Horizontal bars indicate the bootstrap mean; vertical bars indicate the 95% confidence interval.”

Keeping in mind that our power here is limited due to the short no-feedback washout periods, we found that the forgetting rate was consistently close to zero. This is consistent with the idea that motor biases are stable and thus a corrective process to offset the bias (i.e., implicit aiming) would also be stable. The slow forgetting also contrasts with explicit aiming, which, by most accounts, can be turned off within a single trial, resulting in large forgetting rates (>0.1; McDougle & Taylor, 2015).

We have added a supplementary figure (S6) and a discussion section to address these results and their relationship with Forano and Franklin (2020) and Kim et al. (2015) (lines 457-463):

*"We observed a consistently low forgetting rate across the experiments (~0.01, Fig S6). This value is substantially lower than the forgetting rate typically observed when explicit aiming is involved (>0.1)(McDougle et al., 2015; Smith et al., 2006), providing additional evidence that implicit aiming is not a residual component of explicit aiming. More important, the slow rate is in accord with the credit assignment account of implicit aiming. Internal biases are, by nature, assumed to be temporally stable. Given this, a corrective process to offset the bias (i.e., implicit aiming) should also be stable(Forano & Franklin, 2020; Kim et al., 2015).”*

Does the implicit aiming correspond to the new aiming process, also presumably implicit, in Berland et al. 2025? Please discuss.

We have been collaborating with the senior author ([Emmanuel Guigon](https://journals.physiology.org/doi/full/10.1152/jn.00041.2025#con4)) of that paper and have had many discussions on this topic. The model in Berland et al. 2025 is intended to account for implicit recalibration, not implicit aiming. In our new collaborative project, we consider that one part of recalibration is to strengthen the movement trajectory by updating the prediction of a forward model. However, this process does not in itself result in a change in reaching angle. To produce this, there must be a second, inverse-model-like process that generates the appropriate motor output. Together, these two processes produce the behavioral changes arising from implicit recalibration, rather than implicit aiming.

We now cite Berland et al. (2025).

Experiment 2: A detailed comparison showing that there are no differences in aiming errors and target hits during adaptation in the two conditions seems warranted.

Thanks for this suggestion. To address this point, we quantified training performance during adaptation in two ways: aiming error and target hit rate. For each participant, we calculated the mean aiming error and mean hit rate for each of the three training sessions occurring between the aftereffect blocks. We submitted these values to separate 3 × 3 mixed ANOVAs with Condition as a between-subjects factor and Training Session as a repeated-measures factor.

For aiming error, there was a significant main effect of Training Session, F(2,108) = 63.5, p < .001, ηp² = .5. This effect was expected because the perturbation size first increased and then decreased across training, causing the direction of the aiming error to reverse. Critically, there was no main effect of Condition, F(2, 54) = 1.00, p = .38, ηp² = .04, and no Condition × Training Session interaction, F(4, 108) = 0.29, p = .89, ηp² = .01. Thus, although aiming error changed over the course of training, this pattern did not differ across the three conditions.

For target hit rate, there was no main effect of Condition, F(2, 54) = 1.95, p = .15, ηp² = .07, no main effect of Training Session, F(2, 108) = 1.80, p = .17, ηp² = .03, and no Condition × Training Session interaction, F(4, 108) = 1.86, p = .12, ηp² = .07. These results indicate that target hit rate was comparable across the three conditions and did not systematically vary across training sessions.

Together, these analyses show that participants in the three conditions exhibited comparable training performance during adaptation. We have added the corresponding analysis as Fig. S2 and reported the statistics in the Supplementary Materials to support this conclusion.

Line 289. Fig 5F. Typo?

Thanks for noticing this. It should be Fig 3F. It has now been corrected.

Experiment 4: This gradual reduction was included to mimic how "the error signal diminishes in classic visuomotor task". Can the authors explain the rationale for this rather unorthodox choice?

In the original design of Hadjiosif et al. (2023), movement-synchronized feedback was applied under limited reaction time conditions. As such, we would expect implicit aiming and implicit recalibration to both contribute to adaptation. Consequently, even though the perturbation was constant in size, the error signal experienced by the implicit aiming system would not be constant. It would gradually decrease as implicit recalibration progressively offset the perturbation. To account for this, we estimated the "effective" error size driving implicit aiming by first simulating implicit recalibration using a state-space model with a learning rate of 0.05 and retention rate of 0.95 and then subtracting its trial-by-trial contribution from the applied perturbation.

We have clarified this procedure in the Methods section (lines 749-756).

Line 336. "sum the size of the drops" - potentially interesting but vague: what is the sum, and how does it compare? Also need to (re-)justify why this would be the case.

As noted above, we propose that learning in Hadjiosif et al. (2023) involves contributions from both implicit aiming and implicit recalibration. Accordingly, the drop observed after the one-minute break should reflect the combined decay of both processes; this is what we meant by "summing the size of the drops." Hadjiosif et al. (2023) observed a maximum drop in hand angle of approximately 10°, while we observed drops of approximately 5° for each process individually. Thus, the drop reported by Hadjiosif et al. (2023) is consistent with the idea that it is the sum of the two. We recognize limitations with this approach since the error signals driving the two processes cannot be strictly equated when they are measured separately.

The COIN comparison is somewhat questionable. If my understanding is correct, in the COIN simulations of visuomotor adaptation, the main memory update corresponds to the explicit process. The implicit process arises from an updated bias to compensate for residual errors. Please discuss.

As described in the original COIN paper, the full model simultaneously estimates two states: a dynamic state representing the explicit strategic component and a constant state representing an implicit component compensating for systematic motor bias. These two states are additive and are co-modulated by context. We note that the underlying rationale of the COIN model is that while the explicit process dynamically compensates for external perturbations (that change across time), the implicit process compensates for internal bias which presumably is more stable. This is consistent with our interpretation of explicit verse implicit aiming (not implicit recalibration).

We note that the main purpose of simulating the COIN model is to show that the contextual effect observed in implicit aiming is consistent with the modulations predicted by the model. To be clear, how the system attributes error to the implicit and explicit components (i.e., credit assignment) is a separate computational question that the current COIN model does not fully address.

To simulate the dynamics of implicit aiming in Exps 3-4, we applied a COIN model to only estimate the constant state, leaving out the dynamic state. Behavioral change in this model occurs since the estimate of the constant state will evolve across trials.

We have added these details to the modeling section in the Methods (Lines 790-794):

*“The full model simultaneously estimates two states: a dynamic state representing the explicit strategic component and a constant state representing an implicit component that compensates for systematic motor bias. As learning in the Exps 3-4 was largely implicit, we applied a COIN model to only estimate the constant state, leaving out the dynamic state. Behavioral change in this model occurs since the estimate of the constant state will evolve across trials.”*

What is the inset in Figure S1C showing?

The inset in Figure S1C shows performance during the demonstration blocks, the phase used to make sure participants understand the instruction (similar to Fig 2A). The key result here is that the participants showed no-after effect, indicating that they understand the instructions. We have clarified this in the figure legend.
