# Supplementary material for: Implicit sensorimotor adaptation comprises distinct mechanisms for action selection and execution
Source: PLoS Biol. 2026 Oct 9;24(10):e3004057. doi: 10.1371/journal.pbio.3004057 (PMC13667926; doi:10.1371/journal.pbio.3004057)
Supplement: Supplementary file 6 [file pbio.3004057.s013.docx]

**Response to the editor and reviewers**

We thank the editors and reviewers for the positive feedback and constructive suggestions. Per your suggestion, we have changed the title to “Implicit sensorimotor adaptation comprises distinct mechanisms for action selection and execution”.

In accordance with the PLOS Data Policy, we now provide S1 Data (S1_Data.xlsx), which contains the individual numerical values underlying the figures you mentioned.

In accordance with the PLOS Code Policy, we have deposited the code that reproduces the analyses and figures, together with the underlying data, at https://zenodo.org/records/23023244.

We have clarified in the Abstract that the experiments were performed with human participants.

Below, we address the two remaining comments from the reviewer. As both changes are small, we copy the text here and indicate where it appears in the manuscript. We therefore did not provide a separate file with tracked changes.

Reviewer 4:

Reviewer 1 also raised a concern about the magnitude of implicit aiming and implicit recalibration seemingly summing to more than typical total adaptation. The authors' response here is reasoned but essentially argumentative rather than backed by new data — they explain why summing across different isolation paradigms may not be meaningful. I accept this explanation, but would encourage the authors to state in the Discussion that this remains an open question rather than a fully resolved one.

We now explicitly state this as an open question in the Discussion (lines 515–518):

“We note that the relationship between the magnitudes of implicit aiming and implicit recalibration in a natural task remains an open question. In our experiments, each process was measured under conditions designed to isolate it. However, how the two jointly contribute to adaptation when both are engaged, and how they interact with explicit aiming, have yet to be determined.”

One small issue: the authors explain in their response letter (but not, as far as I can tell, in the manuscript itself) why they did not fit the COIN model to Experiments 1-2. I'd suggest adding a brief sentence about this in the manuscript so the omission doesn't look unaddressed to future readers.

We have now clarified this in the Methods (lines 900–905):
“Moreover, we did not simulate Exps 1–2 because the current COIN model does not adequately capture learning under a gradual rotation. The model tends to attribute a gradual perturbation to an external source and to compensate for it through explicit aiming (the dynamic state), resulting in almost no aftereffect, a prediction at odds with the robust aftereffects observed in Exps 1–2 and previous studies. Resolving these issues will require future theoretical work to improve the model's structure.”
