# Supplementary material for: Implicit sensorimotor adaptation comprises distinct mechanisms for action selection and execution
Source: PLoS Biol. 2026 Oct 9;24(10):e3004057. doi: 10.1371/journal.pbio.3004057 (PMC13667926; doi:10.1371/journal.pbio.3004057)
Supplement: Peer review history

# Author comment: Author response to Decision Letter 0

**DOI:** 10.1371/journal.pbio.3004057.r001

1 Jul 2026

Attachment Submitted filename: R2R_PlosBio_26June2026.docx

# Editor report: Decision Letter 0

**DOI:** 10.1371/journal.pbio.3004057.r002

**Authors:** Christian Schnell, PhD

16 Jul 2026

Dear Dr Wang,

Thank you for submitting your manuscript entitled "Distinct Implicit Contributions to Action Selection and Action Execution in Sensorimotor Adaptation" for consideration as a Research Article by PLOS Biology and apologies for the long delay in getting back to you. I was attending a conference last week which delayed our discussion about your revision.

Your manuscript has now been evaluated by the PLOS Biology editorial staff and I am writing to let you know that we would like to send your submission back for external peer review.

However, before we can send your manuscript to reviewers, we need you to complete your submission by providing the metadata that is required for full assessment. To this end, please login to Editorial Manager where you will find the paper in the 'Submissions Needing Revisions' folder on your homepage. Please click 'Revise Submission' from the Action Links and complete all additional questions in the submission questionnaire.

Once your full submission is complete, your paper will undergo a series of checks in preparation for peer review. After your manuscript has passed the checks it will be sent out for review. To provide the metadata for your submission, please Login to Editorial Manager (https://www.editorialmanager.com/pbiology) within two working days, i.e. by Jul 21 2026 11:59PM.

During the process of completing your manuscript submission, you will be invited to opt-in to posting your pre-review manuscript as a bioRxiv preprint. Visit http://journals.plos.org/plosbiology/s/preprints for full details. If you consent to posting your current manuscript as a preprint, please upload a single Preprint PDF.

Feel free to email us at plosbiology@plos.org if you have any queries relating to your submission.

Kind regards,

Christian

Christian Schnell, PhD

Senior Editor

PLOS Biology

cschnell@plos.org

# Author comment: Author response to Decision Letter 1

**DOI:** 10.1371/journal.pbio.3004057.r003

17 Jul 2026

Attachment Submitted filename: R2R_PlosBio_26June2026.pdf

# Peer review: Decision Letter 1

**DOI:** 10.1371/journal.pbio.3004057.r004

**Authors:** Christian Schnell, PhD

14 Sep 2026

Dear Dr Wang,

Thank you for your patience while we considered your revised manuscript "Distinct Implicit Contributions to Action Selection and Action Execution in Sensorimotor Adaptation" for publication as a Research Article at PLOS Biology. This revised version of your manuscript has been evaluated by the PLOS Biology editors, the Academic Editor, one of the original reviewers and a new reviewer to replace Reviewer 1 and Reviewer 3.

Based on the reviews and on our Academic Editor's assessment of your revision, we are likely to accept this manuscript for publication, provided you satisfactorily address the remaining points raised by the reviewers and the following data and other policy-related requests:

* We would like to suggest a different title to improve its accessibility for our broad audience. Would any of the following two suggestions work for you? I'd be happy to discuss other suggestions you may have too.

** Action selection and execution contribute functionally and computationally distinct components to sensorimotor adaption

OR

** Implicit sensorimotor adaptation comprises distinct mechanisms for action selection and execution

* Please add the links to the funding agencies in the Financial Disclosure statement in the manuscript details.

* DATA POLICY:

You may be aware of the PLOS Data Policy, which requires that all data be made available without restriction: http://journals.plos.org/plosbiology/s/data-availability. For more information, please also see this editorial: http://dx.doi.org/10.1371/journal.pbio.1001797

Note that we do not require all raw data. Rather, we ask that all individual quantitative observations that underlie the data summarized in the figures and results of your paper be made available in one of the following forms:

1) Supplementary files (e.g., excel). Please ensure that all data files are uploaded as 'Supporting Information' and are invariably referred to (in the manuscript, figure legends, and the Description field when uploading your files) using the following format verbatim: S1 Data, S2 Data, etc. Multiple panels of a single or even several figures can be included as multiple sheets in one excel file that is saved using exactly the following convention: S1_Data.xlsx (using an underscore).

2) Deposition in a publicly available repository. Please also provide the accession code or a reviewer link so that we may view your data before publication.

Regardless of the method selected, please ensure that you provide the individual numerical values that underlie the summary data displayed in the following figure panels as they are essential for readers to assess your analysis and to reproduce it: 2BE, 4BDE, S1D, S2AB, S5BD and S6.

NOTE: the numerical data provided should include all replicates AND the way in which the plotted mean and errors were derived (it should not present only the mean/average values).

Please also ensure that figure legends in your manuscript include information on where the underlying data can be found, and ensure your supplemental data file/s has a legend.

Please ensure that your Data Statement in the submission system accurately describes where your data can be found.

* CODE POLICY

Per journal policy, if you have generated any custom code during the course of this investigation, please make it available without restrictions. Please ensure that the code is sufficiently well documented and reusable, and that your Data Statement in the Editorial Manager submission system accurately describes where your code can be found. More information on our Code Policy, what and how to share can be found here: https://journals.plos.org/plosbiology/s/code-availability

Please note that we cannot accept sole deposition of code in GitHub, as this could be changed after publication. However, you can archive this version of your publicly available GitHub code to Zenodo. Once you do this, it will generate a DOI number, which you will need to provide in the Data Accessibility Statement (you are welcome to also provide the GitHub access information). See the process for doing this here: https://docs.github.com/en/repositories/archiving-a-github-repository/referencing-and-citing-content

* Please note that per journal policy, the model system/species studied should be clearly stated in the abstract of your manuscript.

As you address these items, please take this last chance to review your reference list to ensure that it is complete and correct. If you have cited papers that have been retracted, please include the rationale for doing so in the manuscript text, or remove these references and replace them with relevant current references. Any changes to the reference list should be mentioned in the cover letter that accompanies your revised manuscript.

In addition to these revisions, you may need to complete some formatting changes, which you will receive in a follow up email. A member of our team will be in touch with a set of requests shortly. If you do not receive a separate email within a few days, please assume that checks have been completed, and no additional changes are required.

We expect to receive your revised manuscript within two weeks.

To submit your revision, please go to https://www.editorialmanager.com/pbiology/ and log in as an Author. Click the link labelled 'Submissions Needing Revision' to find your submission record. Your revised submission must include the following:

- a cover letter that should detail your responses to any editorial requests, if applicable, and whether changes have been made to the reference list

- a Response to Reviewers file that provides a detailed response to the reviewers' comments (if applicable, if not applicable please do not delete your existing 'Response to Reviewers' file.)

- a track-changes file indicating any changes that you have made to the manuscript.

NOTE: If Supporting Information files are included with your article, note that these are not copyedited and will be published as they are submitted. Please ensure that these files are legible and of high quality (at least 300 dpi) in an easily accessible file format. For this reason, please be aware that any references listed in an SI file will not be indexed. For more information, see our Supporting Information guidelines:

https://journals.plos.org/plosbiology/s/supporting-information

*Published Peer Review History*

Please note that you may have the opportunity to make the peer review history publicly available. The record will include editor decision letters (with reviews) and your responses to reviewer comments. If eligible, we will contact you to opt in or out. Please see here for more details:

https://plos.org/published-peer-review-history/

*Press*

Should you, your institution's press office or the journal office choose to press release your paper, please ensure you have opted out of Early Article Posting on the submission form. We ask that you notify us as soon as possible if you or your institution is planning to press release the article.

*Protocols deposition*

To enhance the reproducibility of your results, we recommend that if applicable you deposit your laboratory protocols in protocols.io, where a protocol can be assigned its own identifier (DOI) such that it can be cited independently in the future. Additionally, PLOS ONE offers an option for publishing peer-reviewed Lab Protocol articles, which describe protocols hosted on protocols.io. Read more information on sharing protocols at https://plos.org/protocols?utm_medium=editorial-email&utm_source=authorletters&utm_campaign=protocols

Please do not hesitate to contact me should you have any questions.

Sincerely,

Christian

Christian Schnell, PhD

Senior Editor

cschnell@plos.org

PLOS Biology

------------------------------------------------------------------------

Reviewer remarks

Reviewer #1: This is the revision of a paper I have had previously reviewed. My main complaints were that the original manuscript made it hard for non-expert readers to digest the complex theoretical arguments and succession of experiments and about whether apparent differences between Experiments 1/2 and 3 in inducing perturbations complicated the interpretation of results.

The authors were very responsive to these points. The Venn diagram figure as well as the additional advanced organizer in the Intro and summary in the Discussion considerably increase accessibility. Also the clarifications (in the responses and the manuscript) resolved my issue with the potential procedure differences across experiments.

Overall, the central conclusion rides on the result that delayed feedback indeed abolishes, rather than just reduce implicit calibration. In the light of the absence of any calibration effect in the control condition, I find that result rather convincing. Also, the double-dissociation regarding priming internal-vs-external attribution moving the aftereffect in opposite directions is difficult to explain through the traditional account.

Reviewer #4: This is a revision, and per the editor's request I focused on whether the authors adequately addressed the concerns raised by Reviewer 1 and Reviewer 3.

Overall, I think the authors have done a thorough job.

Reviewer 1's central concern was that "implicit aiming" might simply reflect incompletely suppressed explicit re-aiming rather than a genuine new implicit process. The authors respond with several converging pieces of evidence that are hard to reconcile with this alternative: the instructed practice blocks showing participants can fully switch off an explicit strategy within a single trial, the fact that a simple cover-story manipulation changes the aftereffect 5-fold in a direction opposite to what the "incomplete suppression" account would predict, and the new training-performance analysis (Fig. S2) showing equivalent learning across conditions despite different aftereffects. I find this response convincing.

Reviewer 1 also raised a concern about the magnitude of implicit aiming and implicit recalibration seemingly summing to more than typical total adaptation. The authors' response here is reasoned but essentially argumentative rather than backed by new data — they explain why summing across different isolation paradigms may not be meaningful. I accept this explanation, but would encourage the authors to state in the Discussion that this remains an open question rather than a fully resolved one.

Reviewer 3's main concern was methodological: an incomplete washout in the original Experiment 3. The authors addressed this directly and convincingly by running a new replication with a longer (90-trial) washout, confirming full return to baseline, and replacing the original experiment with this version. This was the right way to handle it. The missing methodological details Reviewer 3 requested (definition of a "cycle," washout rotation, etc.) have also been added.

Reviewer 3's concern about the instructions in Experiments 1-2 being confusing overlaps with Reviewer 1's point above, and is addressed by the same new demonstration-block data and the addition of the full instruction transcript (Appendix 1).

One small issue: the authors explain in their response letter (but not, as far as I can tell, in the manuscript itself) why they did not fit the COIN model to Experiments 1-2. I'd suggest adding a brief sentence about this in the manuscript so the omission doesn't look unaddressed to future readers.

I did not identify any new major technical issues in this revision.

# Author comment: Author response to Decision Letter 2

**DOI:** 10.1371/journal.pbio.3004057.r005

29 Sep 2026

Attachment Submitted filename: R2R_PlosB_28sep26.docx

# Editor report: Decision Letter 2

**DOI:** 10.1371/journal.pbio.3004057.r006

**Authors:** Christian Schnell, PhD

30 Sep 2026

Dear Dr Wang,

Thank you for the submission of your revised Research Article "Implicit sensorimotor adaptation comprises distinct mechanisms for action selection and execution" for publication in PLOS Biology. On behalf of my colleagues and the Academic Editor, Edward K Vogel, I am pleased to say that we can in principle accept your manuscript for publication, provided you address any remaining formatting and reporting issues. These will be detailed in an email you should receive within 2-3 business days from our colleagues in the journal operations team; no action is required from you until then. Please note that we will not be able to formally accept your manuscript and schedule it for publication until you have completed any requested changes.

While you attend to those requests to come, please note that per journal policy, the model system/species studied should be clearly stated in the abstract of your manuscript. Can you add this please, for example by writing "Through a series of studies in human participants..."?

Please take a minute to log into Editorial Manager at http://www.editorialmanager.com/pbiology/, click the "Update My Information" link at the top of the page, and update your user information to ensure an efficient production process.

PRESS

We frequently collaborate with press offices. If your institution or institutions have a press office, please notify them about your upcoming paper at this point, to enable them to help maximize its impact. If the press office is planning to promote your findings, we would be grateful if they could coordinate with biologypress@plos.org. If you have previously opted in to the early version process, we ask that you notify us immediately of any press plans so that we may opt out on your behalf.

We also ask that you take this opportunity to read our Embargo Policy regarding the discussion, promotion and media coverage of work that is yet to be published by PLOS. As your manuscript is not yet published, it is bound by the conditions of our Embargo Policy. Please be aware that this policy is in place both to ensure that any press coverage of your article is fully substantiated and to provide a direct link between such coverage and the published work. For full details of our Embargo Policy, please visit http://www.plos.org/about/media-inquiries/embargo-policy/.

Thank you again for choosing PLOS Biology for publication and supporting Open Access publishing. We look forward to publishing your study.

Sincerely,

Christian

Christian Schnell, PhD

Senior Editor

PLOS Biology

cschnell@plos.org
